# A general approach to the design of allosteric, transcription factor-regulated DNAzymes

**DOI:** 10.1039/c5sc00228a

**Published:** 2015-03-10

**Authors:** G. Adornetto, A. Porchetta, G. Palleschi, K. W. Plaxco, F. Ricci

**Affiliations:** a Dipartimento di Scienze e Tecnologie Chimiche University of Rome Tor Vergata , Via della Ricerca Scientifica , Rome 00133 , Italy . Email: Francesco.ricci@uniroma2.it; b Consorzio Interuniversitario Biostrutture e Biosistemi “INBB” , Rome 00136 , Italy; c Department of Chemistry and Biochemistry , University of California Santa Barbara , Santa Barbara , California 93106 , USA; d Center for Bioengineering , University of California Santa Barbara , Santa Barbara , California 93106 , USA

## Abstract

Here we explore a general strategy for the rational design of nucleic acid catalysts that can be allosterically activated by specific nucleic-acid binding proteins. To demonstrate this we have combined a catalytic DNAzyme sequence and the consensus sequence recognized by specific transcription factors to create a construct exhibiting two low-energy conformations: a more stable conformation lacking catalytic activity and lacking the transcription factor binding site, and a less stable conformation that is both catalytically active and competent to bind the transcription factor. The presence of the target transcription factor pushes the equilibrium between these states towards the latter conformation, concomitantly activating catalysis. To demonstrate this we have designed and characterized two peroxidase-like DNAzymes whose activities are triggered upon binding either TATA binding protein or the microphthalmia-associated transcription factor. Our approach augments the current tool kit for the allosteric control of DNAzymes and ribozymes and, because transcription factors control many key biological functions, could have important clinical and diagnostic applications.

## Abstract 2

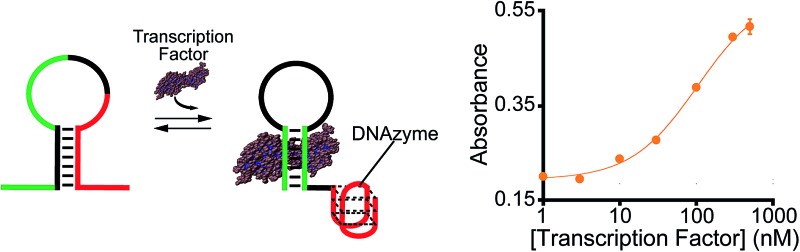
Here we explore a general strategy for the rational design of nucleic acid catalysts that can be allosterically activated by specific nucleic-acid binding proteins.

## Introduction

DNAzymes and ribozymes, naturally occurring or *in vitro* selected RNAs or DNAs^1^ that catalyze specific chemical reactions,^2^–^11^ couple the advantages of enzymes (*e.g.*, high turnover and specificity) with those of nucleic acids (*e.g.*, low cost, ready designability) and thus represent a promising set of tools for use in biosensing,^1^,^12^–^17^ synthetic biology^18^ and bionanotechnology.^1^,^19^–^22^ Catalytic nucleic acids have similarly emerged as a new class of gene silencing agents,^23^,^24^ leading to the development of, for example, new cancer therapies.^25^,^26^

An advantage of nucleic acid catalysts is the ease with which they can be rationally redesigned to introduce allosteric regulation, a mechanism that allows the fine-scale regulation of their activity upon the binding of an effector,^27^ an effect that has proven of value in a range of applications, including synthetic biology^28^–^32^ and biosensing.^33^ Most commonly, the design of allostery has been achieved *via* fusion of the catalytic nucleic acid with a regulation domain for the binding of the effector. This could be a specific short oligonucleotide sequence^34^–^36^ or a small molecule,^37^–^40^ peptide,^33^ or protein^41^ target that binds an aptamer domain. This binding event induces a conformational change that, in turn, regulates the catalytic activity.^28^–^33^,^42^ Here we expand on this theme by demonstrating a new class of allosterically regulated, catalytic nucleic acids that employ nucleic-acid-binding proteins as their effectors.

**Fig. 1:**
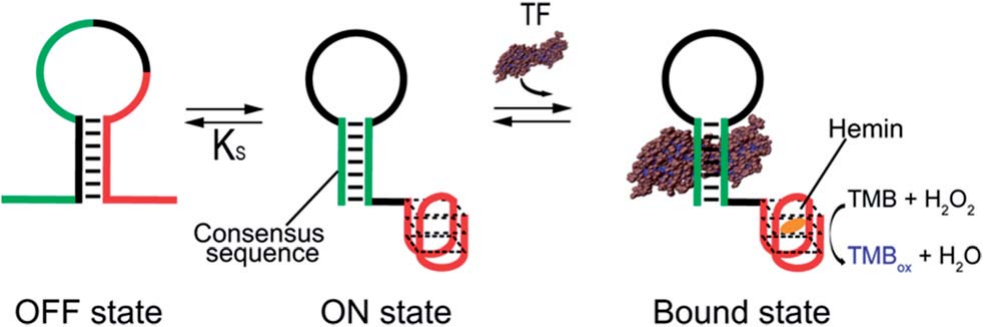
Transcription factor-induced activation of a DNAzyme. Here we demonstrate a DNAzyme allosterically activated by specific transcription factors (TF-regulated DNAzyme). To do so we coupled two functional domains: (i) a catalytic DNAzyme domain (red sequence in the cartoon) and (ii) a double-stranded transcription factor (TF)-binding domain (green). A sequence element complementary to the sequence of the DNAzyme stabilizes an alternative conformation (left) that “sequesters” both domains in an inactive (*i.e.*, non-catalytic and non-TF-binding, respectively) state. This *off-state* is in equilibrium with a second conformation, the *on-state*, in which both domains are functional. TF binding shifts this equilibrium towards the *on-state*, activating catalysis. Here we used the HRP-like G-quadruplex DNAzyme as our model catalytic domain. In the presence of hemin and hydrogen peroxide, this domain catalyzes the oxidation of the HRP substrate 3,3′,5,5′-tetramethylbenzidine (TMB) to give a coloured product which is detectable by absorbance (*λ*_max_ = 650 nm).

As our test bed nucleic-acid catalyst we have employed a guanine-rich, horseradish peroxidase (HRP)-mimicking DNAzyme.^41^,^43^,^44^ This single-strand DNA adopts a G-quadruplex structure that, in the presence of the cofactor hemin, catalyzes the oxidation of HRP-substrates. To re-engineer this DNAzyme to introduce transcription factor (TF)-regulated allostery we combined this catalytic domain (red domain in Fig. 1) with a consensus sequence recognized by a specific TF (green domain in Fig. 1) in such a way that the fusion populates two low-energy conformations. In the more stable of these, termed the *off-state*, the catalytic domain and the double-stranded, TF-binding region are “sequestered” and thus inactive (Fig. 1, left). In the less stable conformation, termed the *on-state*, both the domains are in their functional forms. TF binding thus pushes the equilibrium between these conformations from the former, *off-state*, towards the latter, *on-state*,^45^ activating catalysis (Fig. 1, right).

## Results and discussion

**Fig. 2:**
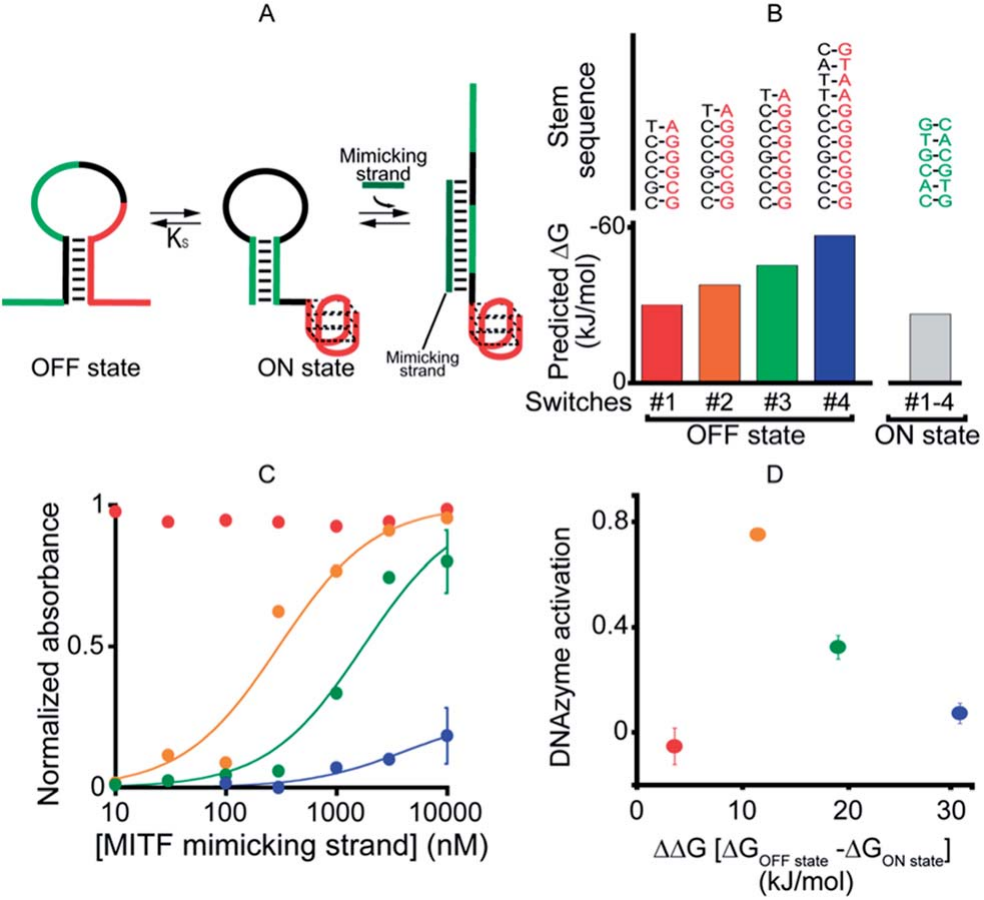
As a first proof-of-principle of transcription factor-induced DNAzyme activation, we designed four DNAzyme variants that are activated by MITF, a transcription factor that is involved in cancer progression. (A) Initially, in order to characterize the different variants, we used a simple DNA strand mimicking the action of the target TF (mimicking strand). (B) In order to modulate the range of the effector's concentration at which activation is observed, the variants were designed with increasingly stable *off-states* whilst maintaining approximately constant stability for the *on-state* (indicated are the *m*-fold predicted free energies). (C) As expected,^45^ the concentration range over which the allosteric effector causes activation of the DNAzyme is dependent on the stability of the *off-state*. (D) Optimal DNAzyme activation is observed at intermediate stabilities of the *off-state* (the *y*-axis shows the DNAzyme activation, the difference in activity when changing the effector concentration from 10 nM to 1 μm). DNAzyme activation was followed here and in the next figures by measuring absorbance at 650 nm in a solution of 50 nM TF-regulated DNAzyme in the presence of the DNAzyme cofactor hemin (500 nM) 40 min after the addition of the DNAzyme substrates TMB and H_2_O_2_. For clarity, error bars have been depicted for only the last point on these binding curves and those in the following figures. These error bars represent the standard deviations of measurements performed on at least three independent replicates.

As our first allosteric effector we employed microphthalmia-associated transcription factor (MITF), a DNA-binding protein associated with melanoma and renal cell carcinoma.^46^–^48^ We designed four variants of MITF-regulated DNAzymes, each presenting the same consensus binding sequence^46^–^48^ for the TF (Fig. 2A, green). The four differ, however, in the stability of their *off-states* (Fig. 2B). Specifically, by increasing the number of bases in the double-stranded stem used to stabilize the *off-state* we obtained variants with estimated^49^ free energies ranging from –30.5 to –57.7 kJ mol^–1^. The stability of the *on-state*, in contrast, is effectively identical in all four variants (predicted stability = –26.9 kJ mol^–1^), and thus this approach provides a means of tuning the switching equilibrium constant, *K*_s_, between the two states. This, in turn, provides a means of controlling the range of TF concentrations over which the DNAzyme is properly regulated.^45^,^50^

To avoid wasting the (rather expensive) TF, we first characterized our re-designed DNAzymes using a DNA strand (the “MITF-mimicking strand”) that binds to and thus stabilizes the *on-state* mimicking the target TF (Fig. 2A). As expected, the range of mimicking-strand concentrations over which activation is observed depends strongly on the switching equilibrium constant (Fig. 2C). Variant #1, for example, which exhibits the least stable *off-state* (predicted Δ*G* = –30.5 kJ mol^–1^), is partially activated even in the absence of its effector. In contrast, variant #4, for which the predicted stability of the *off-state* conformation is the highest (Δ*G* = –57.7 kJ mol^–1^), remains nearly inactive even at the highest effector concentrations we have employed (10 μM). The two variants between these extrema, in contrast, exhibit robustly regulated activation and show the optimal increase in activity upon increasing the effector concentration (Fig. 2D).

**Fig. 3:**
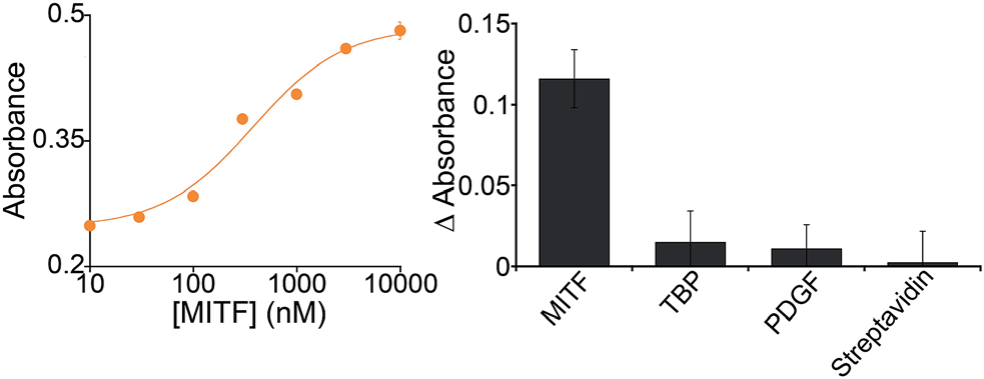
(Left) Our MITF-regulated DNAzyme (variant #2) is activated by its target TF in a dose-dependent fashion and is insensitive to other, non-targeted proteins (right). For the specificity test (right) we have used a concentration of target MITF and of non-specific proteins of 500 nM. In the *y*-axis of the right panel the difference (Δabsorbance) between the absorbance value obtained in the presence of the target and that obtained in a blank solution has been used.

Because it is well regulated, we selected variant #2 to further characterize the extent to which the allosterically regulated catalyst is regulated by its specific TF (Fig. 3). For this variant we observe a monotonic increase in activation with increasing TF and an EC_50_ (the effector concentration at which 50% activation is observed) of 375 ± 105 nM (Fig. 3, left). The activation fold of the DNAzyme activity in the presence of saturating concentration of the target protein is 1.9 which is in good agreement with previous strategies for the allosteric activation of the same G-quadruplex peroxidase-like DNAzyme.^1^,^15^,^27^,^41^

In contrast, the DNAzyme remains inactive when challenged with other, non-effector proteins. Incubation with 500 nM of the transcription factor TATA binding protein, for example, does not produce any significant cross-reactivity (Fig. 3, right).

**Fig. 4:**
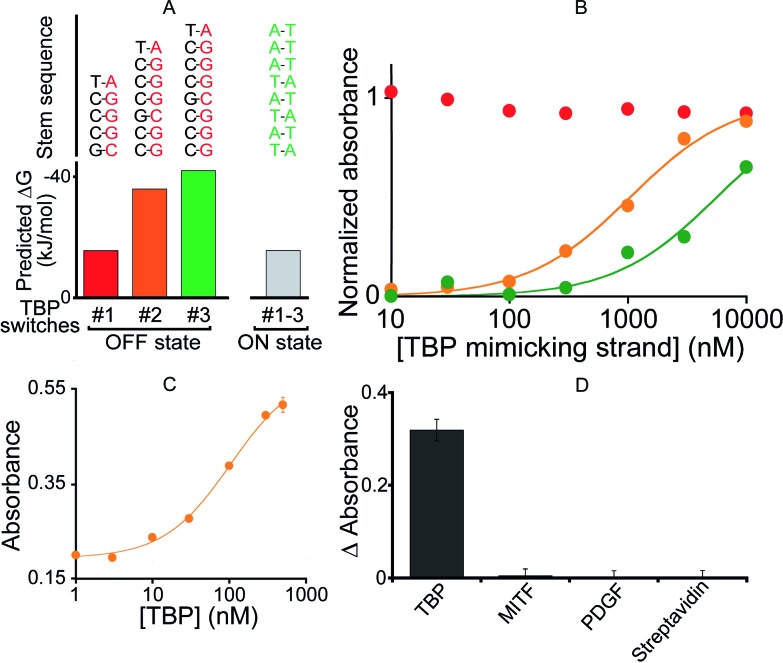
(A) To demonstrate the generality of our strategy we designed variants containing the consensus binding site for TATA binding protein (TBP). (B) Using a DNA strand mimicking the action of the target TF (see Fig. 2a), we find that, as expected, increasing the *off-state* stability shifts the activation profile to higher effector concentrations. (C) The intermediate variant (variant #2) was selected for further tests with the TF showing the activation of the TF-regulated DNAzyme as the concentration of TBP increases (EC_50_ = 104 ± 12 nM). (D) This DNAzyme, too, exhibits good specificity against other non-specific proteins. Here, we have used a concentration of target TBP and of non-specific proteins of 500 nM. In the *y*-axis of panel D the difference (ΔAbsorbance) between the absorbance value obtained in the presence of the target and that obtained in a blank solution has been used.

To demonstrate the generality of our approach we designed a DNAzyme that is, instead, regulated by TATA binding protein (TBP), a TF present in virtually all eukaryotic cells.^51^ To do so, we engineered three variants differing in *K*_s_ (Fig. 4A and B) that, upon switching to the *on-state*, exhibit the consensus binding sequence of TBP.^50^,^51^ We found that the resultant activation profiles (using again a TF-mimicking DNA strand) are consistent with the predicted energy gap between the *on*- and *off-states*. From these results we selected variant #2, which exhibits an intermediate *K*_s_ and thus robust activation, for further characterization. The 2.6-fold, TBP-induced activation of this DNAzyme occurs with an EC_50_ of 104 ± 12 nM (Fig. 4C) and, as was true for its MITF-activated counterpart, the DNAzyme is insensitive to other, non-targeted proteins (500 nM) (Fig. 4D).

## Conclusions

In nature, the regulation of the activity of an enzyme is usually achieved through allosteric regulation, in which a binding site distal from the active site can recognize a molecule (an allosteric effector) and lead to a conformational switch that affects the enzyme's affinity for the substrate. Through this mechanism the activity of naturally occurring enzymes can be finely regulated by a variety of allosteric effectors in a concentration-dependent fashion. Allostery, also called “the second secret of life” by Perutz,^52^ can thus be considered as the optimal strategy to regulate the affinities and activities of biomolecules and bioreceptors. Because of this, engineering allostery into artificial systems could greatly improve the functionality of biomolecules employed in biotechnologies. In response, we have demonstrated here a general strategy for the design of nucleic acid catalysts allosterically activated by specific transcription factors.

The development of allosterically regulated DNAzymes or ribozymes through both rational design and combinatorial selection strategies has been the subject of several studies starting from the seminal and fundamental work of Breaker and coworkers regarding the design and development of allosteric ribozymes.^2^,^3^,^8^,^37^,^53^–^55^ In the past ten years several efforts have been devoted to the development of nucleic acid catalysts that can be activated or regulated by different effectors including short nucleic acid sequences,^21^,^34^–^36^ and aptamer targets.^33^,^37^–^41^ Allosteric nucleic acid catalysts have thus been demonstrated to be useful tools not only for biosensing purposes^56^ but also as controllable therapeutic agents for gene therapy strategies^23^–^26^ or as molecular tools for controlling gene expression.^30^–^32^ Despite this, to the best of our knowledge, there is no report of a rational design of DNAzymes allosterically regulated by transcription factors (TF). We demonstrated here a general strategy to obtain TF-responsive DNAzymes by joining a TF consensus sequence with a G-quadruplex peroxidase-like DNAzyme domain. Our approach is based on the rational design of a conformational switching probe that flips between a non-binding inactive conformation to a second binding-competent active conformation in the presence of the specific TF. Because of this, we can regulate the TF concentration range at which activation of the DNAzyme is observed. We can thus control in a fine-tuned fashion the activity of our TF-regulated DNAzyme not only using different concentrations of the specific TF, but also using variants with different stabilities of the non-binding conformation, over completely different TF concentration ranges.

Compared to similar strategies reported earlier on the allosteric activation of catalytic nucleic acids, our approach appears very versatile. The consensus site recognized by each TF almost invariably consists of a specific double stranded DNA sequence with a length ranging from 6 to 12 base pairs, rendering the rational design of the necessary conformational switch quite straightforward. We also note that our approach can have important biological and clinical implications. While there is only a limited number of molecular targets recognizable *via* the use of aptamers, more than 10% of the *ca.* 25 000 human genes encode DNA-binding proteins suitable for the regulatory role presented here, the majority of which function as transcription factors thus controlling crucial biological mechanisms.^57^ Despite the limited activation fold achieved with our strategy, we note that this is comparable to other examples of allosterically activated DNAzymes based on the use of the peroxidase-like G-quadruplex.^1^,^15^,^27^,^41^ Because other examples of DNAzymes and ribozymes activated by different targets show larger activation folds we believe that this difference might be due to the low efficiency and high background signal characteristics of peroxidase-like DNAzyme in comparison to other DNAzymes.^1^,^27^,^42^–^44^ Finally, our strategy could be applied in the design of RNA/DNA chimeras to produce TF-regulated ribozymes and RNA-binding-protein-regulated DNAzymes that could have potential applications in several fields. Engineering allosteric catalytic nucleic acids that respond to TFs could in fact lead to novel molecular tools for gene expression regulation, gene regulatory mechanisms or novel riboregulators that can allow the development of genetic circuits in the growing fields of synthetic biology and bioengineering.^58^–^60^

## Experimental section

Unmodified oligonucleotides were synthesized and purified (desalt purification) either by IBA GmBH (Göttingen, Germany) or Sigma-Aldrich (St. Louis, Missouri) and used without further purification. The sequences of the constructs are as follows.

MITF variant #1: 5′-C*CACGTG* CGCCCT *CACGTG* TCTTGGGT AGGGCG GGTTGGG-3′.

MITF variant #2: 5′-C*CACGTG* CCGCCCT *CACGTG* TCTTGGGT AGGGCGG GTTGGG-3′.

MITF variant #3: 5′-C*CACGTG* CCCGCCCT *CACGTG* TCTTGGGT AGGGCGGG TTGGG-3′.

MITF variant #4: 5′-C*CACGTG* CCCGCCCTA *CACGTG* TCTTGG GTAGGGCGGG TTGGG-3′.

TBP variant #1: 5′-C*TATATAAA* GCCCT *TTTATATA* TCTTGGGT AGGGC GGGTTGGG-3′.

TBP variant #2: 5′-C*TATATAAA* CCGCCCT *TTTATATA* TCTTGGGT AGGGCGG GTTGGG-3′.

TBP variant #3: 5′-C*TATATAAA* GCCCGCCCT *TTTATATA* TCTTGGGT AGGGCGGG TTGGG-3′.

In the sequences above the underlined bases represent the stem portion of the non-binding conformation (*off-state*), while the italic bases represent the TF recognition element of the binding conformation (*on-state*) (see Fig. 1).

Mimicking strand oligonucleotides were purchased either from IBA GmBH (Göttingen, Germany) or from Sigma-Aldrich (St. Louis, Missouri) and below their sequences are reported:

MITF-mimicking strand: 5′-AGACAC GTGAGG-3′.

TBP-mimicking strand: 5′-AGATATA TAAAAGG-3′.

The mimicking strand sequences bind the TF-regulated DNAzyme sequences in a manner that induces the TF-regulated DNAzyme to adopt its catalytically active conformation.

Microphthalmia-associated transcription factor (MITF) was a gift from Prof. Colin Goding's group at the University of Oxford (UK). It is constituted by residues 198–302 of the human MITF-M (melanocyte specific), which encompasses the DNA-binding domain bHLH-LZ.^48^

TATA-binding protein was obtained by expression of recombinant, His-tagged proteins in *Escherichia coli*, as described previously.^61^

All experiments with TFs and mimicking strands were conducted with a DNAzyme concentration of 50 nM in a 25 mM HEPES buffer containing 20 mM KCl, 200 mM NaCl and 1% DMSO at pH 7.4. Absorbance at 650 nm was measured 40 min after the addition of the DNAzyme substrates, 3,3′,5,5′-tetramethylbenzidine (TMB) liquid substrate, supersensitive, for ELISA (ready-to-use solution with H_2_O_2_, purchased from Sigma-Aldrich, St. Louis, Missouri) using either a Bio-Rad Model 550 Microplate Reader or a Tecan Infinite M1000 PRO.

Activation curves were measured in microtiter plates. In every well, a concentration of the TF-regulated DNAzyme of 50 nM was used in a total volume of 150 μl and different concentrations of the proper mimicking strand or protein were added and allowed to react for 1 h at 37 °C. After this, the DNAzyme cofactor hemin was added at a final concentration of 500 nM and incubated for 1 h at 37 °C. Finally, 150 μl of the DNAzyme substrates TMB and H_2_O_2_ were added and the absorbance was measured at 650 nm after 40 min of incubation. The absorbance of the *off-state* was set relative to 0 for all normalized figures, while the absorbance obtained in the presence of a saturating target concentration was set relative to 1. The curves were fitted with the following equation:
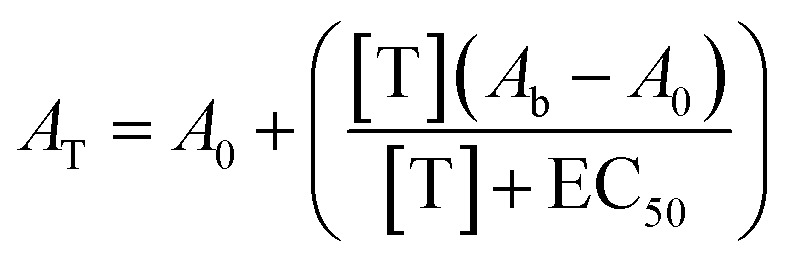
where *A*_T_ is the absorbance in the presence of a different concentration of target; *A*_0_ is the background absorbance; [T] is the concentration of the TF or the mimicking strand; *A*_b_ is the absorbance in the presence of a saturating concentration of target; and EC_50_ is the target concentration at which the activation is half of the maximum activation.
